# KLF4, a miR-32-5p targeted gene, promotes cisplatin-induced apoptosis by upregulating BIK expression in prostate cancer

**DOI:** 10.1186/s12964-018-0270-x

**Published:** 2018-09-03

**Authors:** Lu Zhang, Xiaojie Li, Yulin Chao, Ruiping He, Junqiang Liu, Yi Yuan, Wenzhi Zhao, Chuanchun Han, Xishuang Song

**Affiliations:** 1 Department of Urology of the First Affiliated Hospital & Institute of Cancer Stem Cell, Dalian Medical University, Dalian, Liaoning 116044 People’s Republic of China; 2 Department of Orthopedics, Second Affiliated Hospital, Dalian Medical University, Dalian, 116044 China; 3 College of Stomatology, Dalian Medical University, Dalian, 116044 China

## Abstract

**Background:**

Chemotherapeutic insensitivity remains a big challenge in prostate cancer treatment. Recently, increasing evidence has indicated that KLF4 plays a key role in prostate cancer. However, the potential biological role of KLF4 in Chemotherapeutic insensitivity of prostate cancer is still unknown.

**Methods:**

The role of KLF4 in cisplatin-induced apoptosis was detected by western blotting and a cell counting kit (CCK8). The potential molecular mechanism of KLF4 in regulating prostate cancer chemosensitivity was investigated by RNA sequencing analysis, q-RT-PCR, western blotting and chromatin immunoprecipitation (ChIP). The expression level of KLF4 mediated by miR-32-5p was confirmed by bioinformatic analysis and luciferase assays.

**Results:**

Here, we found that KLF4 was induced by cisplatin in prostate cancer cells and that the increase in KLF4 promoted cell apoptosis. Further mechanistic studies revealed that KLF4 directly bound to the promoter of BIK, facilitating its transcription. Additionally, we also found that the gene encoding KLF4 was a direct target of miR-32-5p. The downregulation of miR-32-5p in response to cisplatin treatment promoted KLF4 expression, which resulted in a increase in the chemosensitivity of prostate cancer.

**Conclusion:**

Thus, our data revealed that KLF4 is an essential regulator in cisplatin-induced apoptosis, and the miR-32-5p-KLF4-BIK signalling axis plays an important role in prostate cancer chemosensitivity.

**Electronic supplementary material:**

The online version of this article (10.1186/s12964-018-0270-x) contains supplementary material, which is available to authorized users.

## Background

Prostate cancer (PC) is one of the most aggressive malignant cancers and is the third-leading cause of death from cancer in men [1]. Although androgen deprivation therapy through either chemical or surgical castration initially works well to control metastatic prostate cancer, all patients eventually progress to castration resistant prostate cancer, for which no effective treatment is currently available [2–5]. Chemotherapy can only prolong patient survival by a few months in castration-resistant disease due to its insensitivity to conventional chemotherapies, resulting in tumour recurrence [6–8]. Therefore, understanding the molecular mechanisms of Chemotherapeutic insensitivity is crucial to develop effective therapeutic strategies for prostate cancer.

KLF4/GKLF is a member of the KLF-like factor subfamily of zinc finger proteins [9]. Dysregulation of KLF4 has been observed in a number of human cancers, including gastrointestinal, pancreas, bladder, and lung cancer. Ectopic expression of KLF4 has been reported to suppress cell proliferation, induce apoptosis, and promote cell-cycle arrest, indicating that KLF4 has a tumour suppressor function in a variety of malignancies and its downregulation may play an essential role in tumourigenesis [10–15]. However, in squamous cell carcinoma, breast cancer and osteosarcoma, KLF4 was shown to promote cell growth, cellular dedifferentiation and inhibit cell apoptosis [10, 16, 17]. Thus, the ability of KLF4 to act as either a tumour suppressor or an oncogene is largely dependent on tissue type, tumour type and tumour stage. In prostate cancer, the expression level of KLF4 has been shown to be downregulated. Overexpression of KLF4 inhibited prostate cancer cell growth and metastasis [18, 19]. Although KLF4 was found to be a tumour suppressor in prostate cancer, the effect of KLF4 on Chemotherapeutic insensitivity is still unknown.

In this study, we found that KLF4 expression was induced by cisplatin in prostate cancer cells and increased levels of KLF4 promoted cell apoptosis. Further mechanistic studies revealed that KLF4 was directly bound to the promoter of BIK, facilitating its transcription. Additionally, we also found that the gene that encodes KLF4 was a direct target of miR-32-5p. The downregulation of miR-32-5p in response to cisplatin treatment promoted an increase in KLF4 expression and in the sensitivity of prostate cancer to cisplatin. Thus, our data revealed that KLF4 is an essential regulator in cisplatin-induced apoptosis, and the miR-32-5p-KLF4-BIK signalling axis plays an important role in prostate cancer chemoresistance.

## Methods

### Cell culture and reagents

PC-3 and DU145 cells were maintained in RPMI-1640 medium supplemented with 10% foetal bovine serum FBS (ExCell Bio, Lot: FSP500), 2 mM L-glutamine, penicillin (100 U/ml), streptomycin (100 μg/ml) and 0.1% Savelt ™ (Hanbio Co. LTD 1:1000) in a humidified atmosphere of 5% CO_2_ maintained at 37 °C. The following antibodies were used in this study: antibodies against GAPDH (Santa Cruz Biotechnology, Dallas, TX, USA; SC-25778, 1:1000), PARP (Santa Cruz Biotechnology, SC-8007, 1:1000), KLF4 (Santa Cruz Biotechnology, SC-20691, 1:10 for ChIP), KLF4 (Cell Signaling Technology, #12173S, 1:500), and BIK (Abcam, ab52182, 1:500).

### RNA Interference and KLF4 knockout cell generation

RNA interference was performed as previously described [20]. The sequences targeting BIK-1 were: 5-CTTCGATTCTTTGGAATGCAT-3 and BIK2–2 5-CCACACTTAAGGAGAACATAA-3; KLF4–1, 5-ATCGGTCATCAGCGTCAGCAA-3; KLF4–2 5-AAGTCATCTTGTGAGTGGATAA-3.

KLF4 knockout by CRISPR/Cas9: sgRNA design and cloning was performed according to the Feng Zhang laboratory general cloning protocols. KLF4 sgRNAs oligonucleotides were designed based on the target site sequence (20 bp) and were flanked on the 3′ end by a 3 bp NGG PAM sequence. Using the Cas9 target design tools (http://www.genome-engineering.org), we designed two sgRNAs for each target: KLF4 sg1 UP: 5-CACCCGCCGGGCCAGACGCGAACG-3, DN: 5-AAACCGTTCGCGTCTGGCCCGGCG-3; Sg2 UP: 5-CACCGTCTTTCTCCACGTTCGCGTC-3, DN: 5-AAACGACGCGAACGTGGAGAAAGAC-3. The sgRNAs were cloned into the lentiCRISPRv2 vector (Addgene). For lentivirus production, cloned lentiCRISPRv2 plasmids were co-transfected into HEK293T cells with the packaging plasmids pVSVg (AddGene 8454) and psPAX2 (AddGene 12260). The lentivirus was harvested. PC3 and DU145 cells were infected with the two sgRNA mixtures for KLF4. Forty-eight hours after infection, cells were placed under puromycin selection for 2 weeks and the single-cell-derived clones were picked, expanded and knockout of KLF4 was verified by western blotting analysis.

### Cell transfections and virus infection

Prostate cancer cells were transfected with indicated plasmids using Lipofectamine 3000 (Invitrogen) reagent according to the manufacturer’s protocol. To generate lentivirus expressing KLF4 or BIK, HEK 293 T cells grown on a 6 cm dish were transfected with 2 μg pCDH-KLF4 or pCDH-BIK or control vector (pCDH), 1.5 μg psPax2, and 0.5 μg pMD2G. 24 h after the transfection, cells were cultured with DMEM containing 10% FBS for an additional 24 h. The culture medium containing lentiviral particles was centrifuged at 1,000 g for 5 min. Viral particles collected in the supernatant were used for infection. In order to establish the stable cell line, the puromycin was used as a selection marker for the infected cells. The expression efficiency was evaluated by western blot analysis.

### Cell viability assay

Cells were plated in 96-well plates at a density of 800 cells in 200 μl of medium per well 24 h before the experiment. Following treatments, the cell viability was determined using a CCK8 kit (Cell Counting Kit-8).

### Annexin V-FITC staining and FACS

The staining protocol was performed following the manufacturer’s instructions (BD). Generally, prostate cancer cells (5 × 10^5^) treated as indicated were harvested by a 5 min centrifugation at 1000 *g* and resuspended in 195 μL binding buffer, followed by a 10 min incubation with 5 μL Annexin V-FITC at room temperature avoiding any light. After an additional centrifugation, the cells were resuspended in 190 μL binding buffer and 10 μL PI stain was added with slight shaking. FACS (BD) analysis was employed for detecting cell apoptotic events.

### ChIP assay

The ChIP assay was performed as previously described [21].

Quantitative real-time polymerase chain reaction assay (q-RT-PCR).

Total RNA was isolated using TRIzol (Invitrogen). One microgram of total RNA was used to synthesize cDNA using the PrimeScript™ RT reagent kit (Takara, RR047A) according to the manufacturer’s instructions. The primers were as follows: BIK UP: 5-GACCTGGACCCTATGGAGGAC-3, DN: 5-CCTCAGTCTGGTCGTAGATGA-3; and ACTIN UP: 5-CACCTTCTACAATGAGCTGCGTGTG-3, DN: 5-ATAGCACAGCCTGGATAGCAACGTAC-3; KLF4 UP: 5-ACCTACACAAAGAGTTCCCATC-3, DN: 5-TGTGTTTACGGTAGTGCCTG-3. The primers for mature miR-32-5p were purchased from Takara.

### Promoter reporters and dual-luciferase assay

The upstream sequence of BIK and different truncations were inserted into pGL3-based luciferase reporter plasmids. The followed primers were used for PCR. UP: 5- GCGGTACC ACCCAACAGGTAGCAA-3, DN:5- GCCTCGAGGGCCCGGCTGCCGGCGC-3 for P1; UP: 5- GCGGTACC ACCCAACAGGTAGCAA-3, DN: 5- GCCTCGAGACAAATATGAAAACCGAGG-3 for P2; UP: 5- GCGGTACC GAAATAGGCTTTAAAACA-3, DN: 5-GCCTCGAGGGCCCGGCTGCCGGCGC-3 for P3. The sequence of KLF4 3’UTR was cloned into pSICHECK2 vector. The followed primers were used for PCR: UP: 5-GCCTCGAG ATCCCAGACAGTGGATAT-3. DN: 5-GCGCGGCCGC TTCAGATAAAATATTAT-3. The plasmids were transfected into osteosarcoma cells and after transfection, luciferase activity was measured in a 1.5-ml Eppendorf tube with the Promega Dual-Luciferases Reporter Assay kit (Promega E1980) according to the manufacturer’s protocols. Relative Renilla luciferase activity was normalized to firefly luciferase activity. The assay was performed as previously described [22, 23].

### Introduction of microRNA mimics and inhibitors

Mimics and inhibitors of miRNA-32-5p were synthesized by the GenePharma Company (Shanghai, People’s Republic of China). For each transfection in a six-well plate, 100 nM miRNA mimics, mimic control or inhibitor, or inhibitor control were used. The transfection of prostate cancer cells by Oligofectamine (Invitrogen) was performed according to the manufacturer’s instructions.

### Statistics and data analyses

Data are expressed as the mean ± SD, and statistical evaluation was performed using one-way analysis of variance (ANOVA). Values of *p* < 0.05 were considered statistically significant.

## Results

### KLF4 promotes cisplatin-induced apoptosis in prostate cancer

**Fig. 1:**
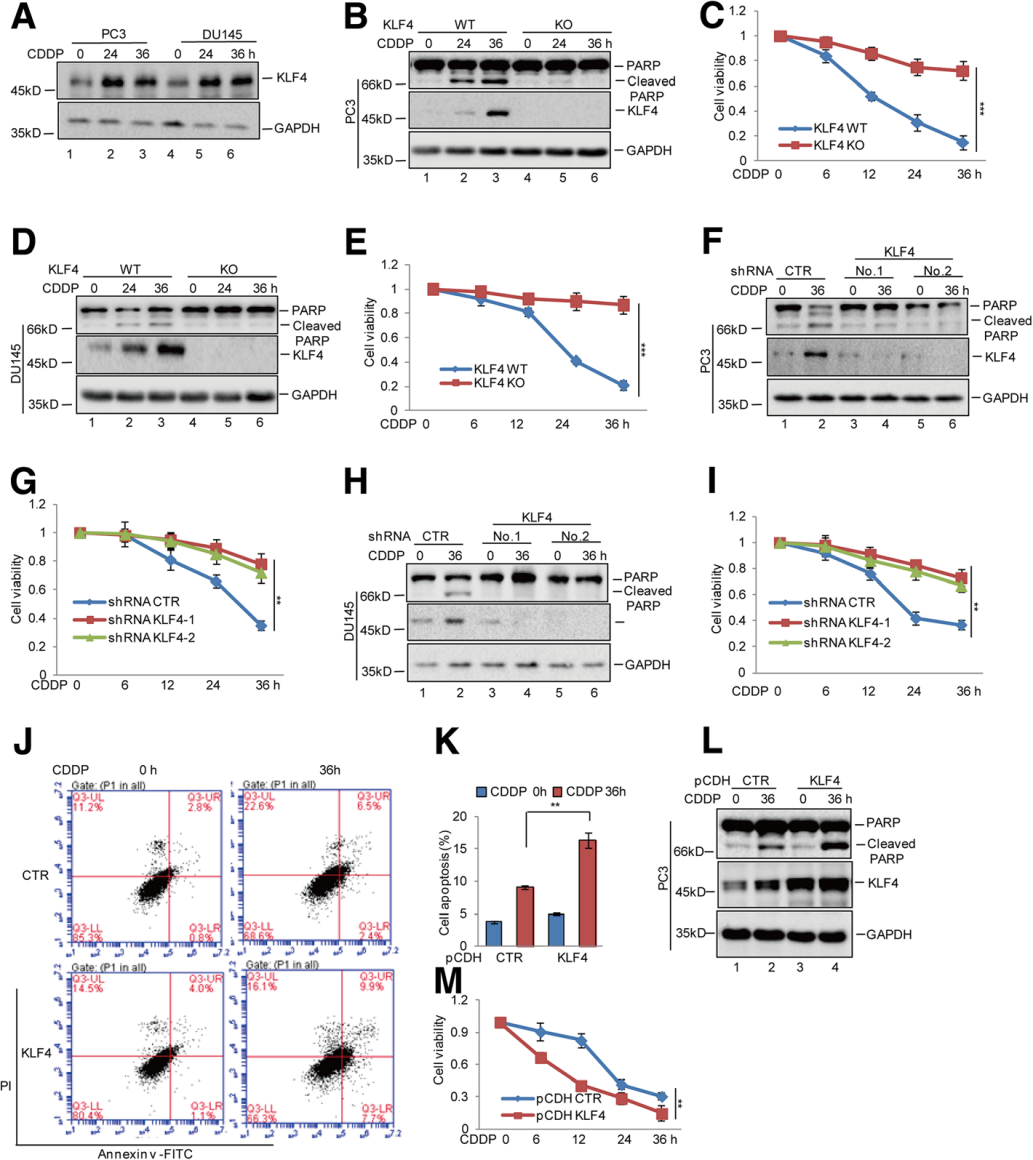
KLF4 enhanced cisplatin-induced apoptosis in prostate cancer cells. **a** PC3 and DU145 cells were treated with 20 μM cisplatin (CDDP) at the indicated times. The protein levels of KLF4 were analysed by western blotting. **b**-**e** PC3 and DU145 cells with or without KLF4 knockout (KO) were treated with 20 μM cisplatin at the indicated times. Cell apoptosis was detected by western blotting. Cell viability was measured by a CCK8 assay. Data represent the mean ± SD of three independent experiments. ***p < 0.001 vs. control. **f**-**i** PC3 and DU145 cells with or without KLF4 knockdown were treated with 20 μM cisplatin at the indicated times. Cell apoptosis was detected by western blotting and cell viability was measured by a CCK8 assay. Data represent the mean ± SD of three independent experiments. **p < 0.01 vs. control. **j**-**m** PC3 cells with or without KLF4 overexpression were treated with 20 μM cisplatin as indicated. Cell apoptosis was analysed by flow cytometer and western blotting. Cell viability was detected by a CCK8 assay. Data represent the mean ± SD of three independent experiments. **p < 0.01 vs. control

To investigate the role of KLF4 in Chemotherapeutic insensitivity of prostate cancer, we first analysed the expression level of KLF4 under cisplatin (CDDP) treatment and found that KLF4 expression was increased in response to cisplatin (Fig. 1a). To further assess the effect of increased KLF4 on cisplatin-induced apoptosis, we first knocked out KLF4 in PC3 and DU145 cells using CRISPR/Cas9 technology and treated the cells using cisplatin. Interestingly, we found that KLF4 knockout (KO) strongly reduced apoptosis, as indicated by a PARP cleavage decrease and elevated cell viability in prostate cancer cells (Fig. 1b-e). To further confirm this, we then knocked down KLF4 expression with 2 independent shRNAs in PC3 and DU145 cells. As shown in Fig. 1f-i, inhibition of KLF4 significantly decreased cell apoptosis and increased cell viability. Conversely, escalating apoptosis and diminishing cell viability was observed in prostate cancer cells overexpressing exogenous KLF4 (Fig. 1j-m).

### KLF4 upregulates BIK expression during cisplatin treatment

**Fig. 2:**
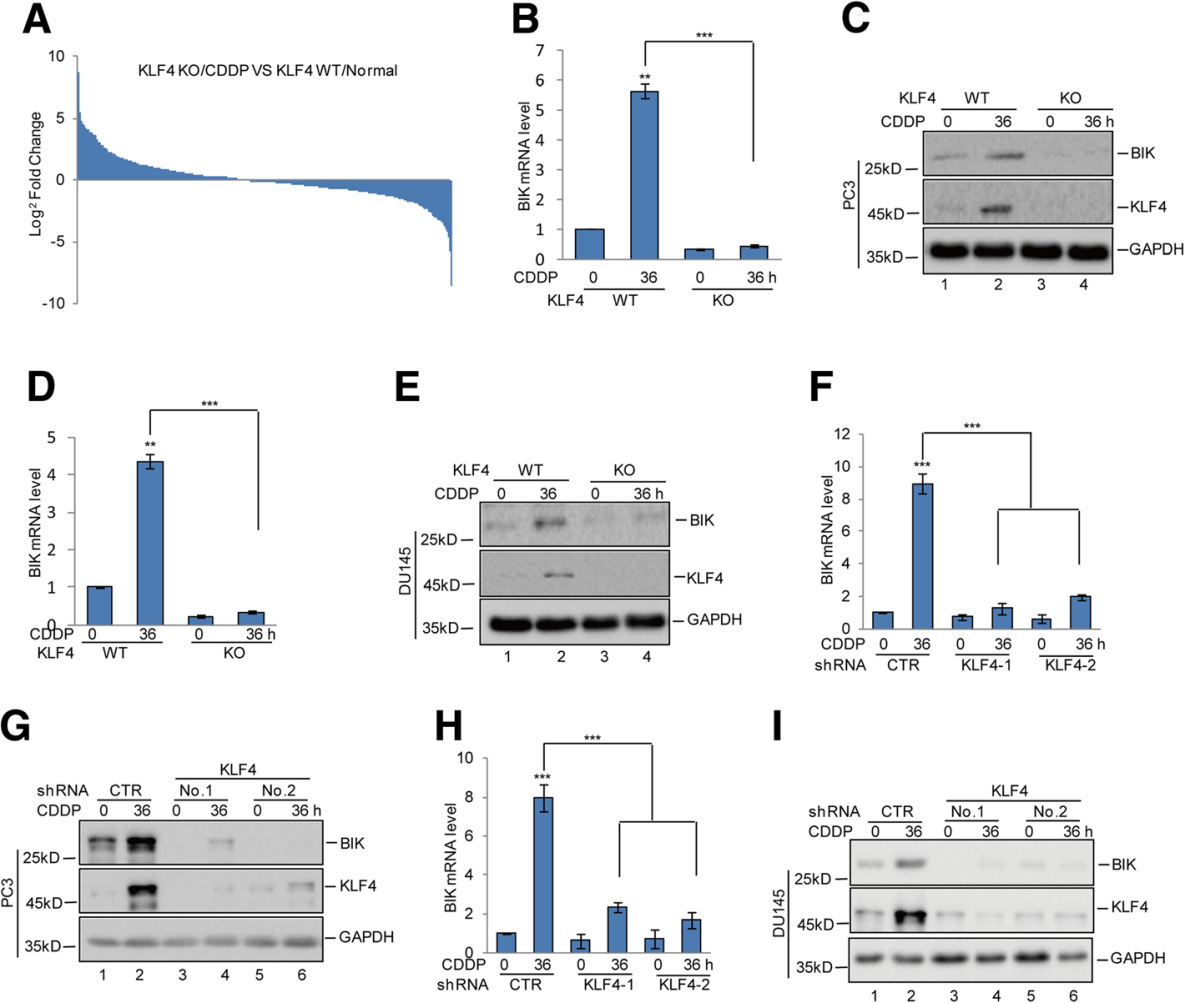
KLF4 upregulated BIK expression. **a** KLF4 WT or KO PC3 cells were treated with 20 μM cisplatin at the indicated times. Gene expression profiles were obtained by RNA sequencing analysis. **b**-**e** PC3 and DU145 cells with or without KLF4 KO were treated with 20 μM cisplatin at the indicated times. The mRNA and protein levels of BIK were analysed by q-RT-PCR and western blotting. Data represent the mean ± SD of three independent experiments. *** *p* < 0.001 vs. control. **f**-**i** PC3 and DU145 cells with or without KLF4 knockdown were treated with 20 μM cisplatin at the indicated times. The mRNA and protein levels of BIK were analysed by q-RT-PCR and western blotting. Data represent the mean ± SD of three independent experiments. ***p < 0.001 vs. control

To uncover the molecular mechanism underlying the regulation of cisplatin-induced apoptosis by KLF4, gene expression profiles in KLF4 wild type (WT) and KO PC3 cells with or without cisplatin treatment were obtained by RNA sequencing analysis (Fig. 2a and Additional file 1: Figure S1A-1C). Among the altered genes, we focused on the genes that were involved in regulating cell apoptosis. We found that KLF4 KO significantly decreased BIK upregulation in response to cisplatin treatment, which was subsequently confirmed using quantitative PCR and western blotting analysis in PC3 and DU145 cells (Fig. 2b-e). To further prove the effects of KLF4 on BIK expression, PC3 and DU145 cells with or without KLF4 knockdown were treated with cisplatin and the expression level of BIK was analysed. Similarly, we found inhibition of KLF4 expression suppressed the BIK increase (Fig. 2f-i).

### KLF4 directly binds to the promoter of BIK

**Fig. 3:**
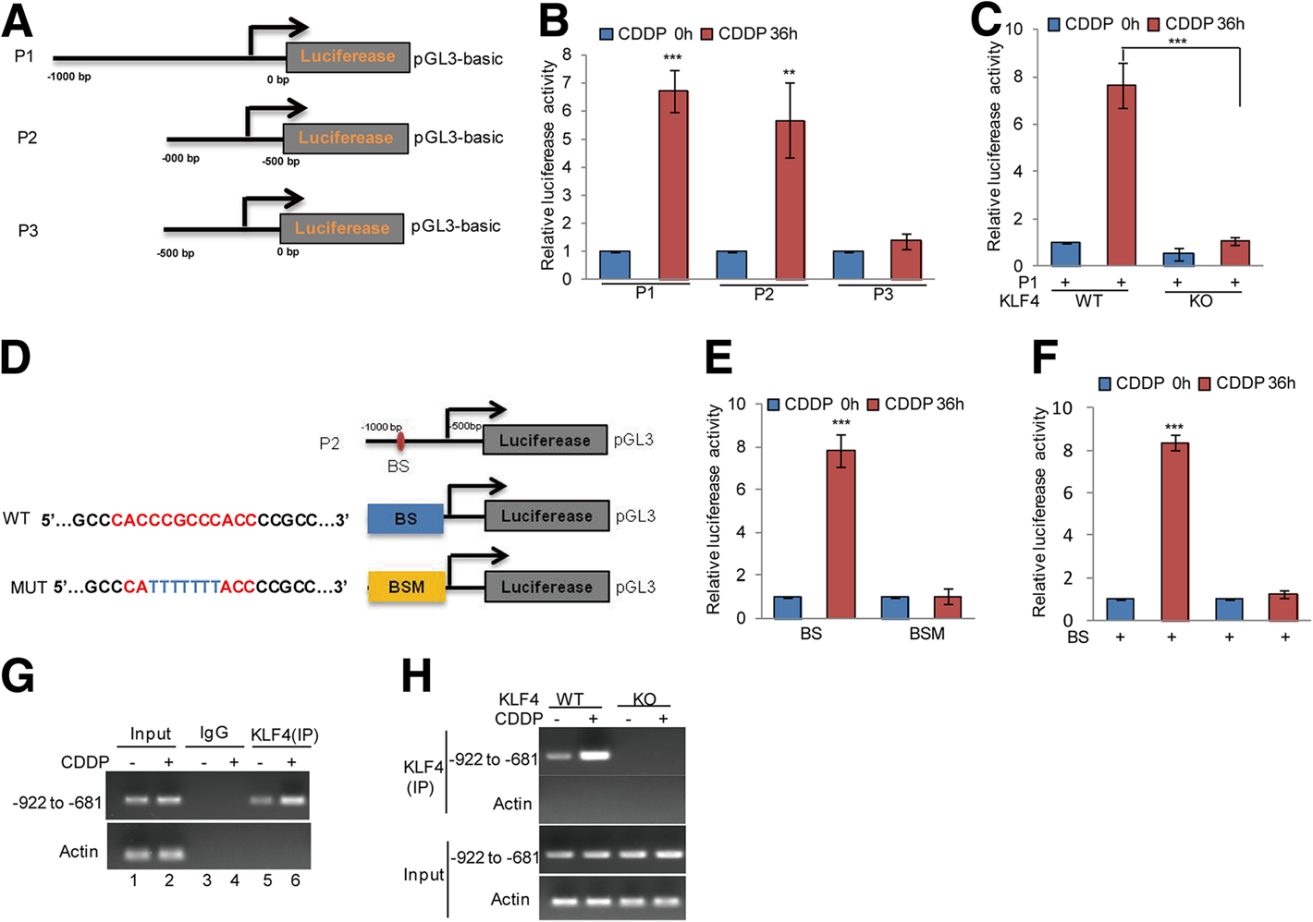
KLF4 bound to the promoter of BIK. **a** Schematic illustration of pGL3-based reported constructs were used in luciferase assays to examine the transcriptional activity of BIK. **b** Parts of the promoter of BIK, named P1, P2 and P3, were individually transfected into PC3 cells with or without 20 μM cisplatin treatment. Luciferase activity was measured. Data represent the mean ± SD of three independent experiments. **p < 0.01, *** *p* < 0.001 vs. control. **c** The promoter of BIK, named P1, was transfected into PC3 and DU145 cells with or without KLF4 KO and then the cells were treated with 20 μM cisplatin at the indicated times. Luciferase activity was measured. Data represent the mean ± SD of three independent experiments. ***p < 0.001 vs. control. **d** The potential KLF4 binding sites were inspected by JASPAR. Schematic illustration of KLF4 wild type binding site (BS) and the matching mutant (BSM) that were used in luciferase assays. **e**-**f** The wild type promoter (BS) or the matching mutant (BSM) were individually transfected into PC3 cells with or without KLF4 knockout and the cells then were treated with 20 μM cisplatin for the indicated times. Luciferase activity was measured. Data represent the mean ± SD of three independent experiments. *** *p* < 0.001 vs. control. **g**-**h** ChIP analysis showing the binding of KLF4 to the promoter of BIK in KLF4 WT or KO PC3 cells in response to 20 μM cisplatin treatment. An isotype-matched IgG was used as a negative control

To identify the KLF4 binding regions on the BIK promoter, we first cloned the upstream sequence of BIK and different truncations by PCR. Then, we inserted them into pGL3-based luciferase reporter plasmids, named P1–P3 (Fig. 3a). We transfected them into PC3 cells with or without cisplatin treatment. As shown in Fig. 3b, the luciferase activities of P1 and P2 were augmented in PC3 cells exposed to cisplatin treatment; however, this increase was abolished when P2 was transfected, indicating that the region − 1000 to − 500 bp was a key region for the promotion of BIK under cisplatin treatment (Fig. 3b). To further verify whether the region was essential for KLF4, these truncations were transfected into PC3 cells with or without KLF4 KO. We found that cisplatin induced an increase of luciferase activity from P1. However, this increase disappeared when KLF4 was knocked out (Fig. 3c).

Previous reports have indicated that KLF4 is a zinc finger-type transcription factor that usually binds to the GC rich element of the promoters [24]. To identify the potential KLF4 binding sites, we inspected the sequence of the BIK promoter by JASPAR software and found a putative KLF4 binding site on the KLF4 promoter. To verify that the potential KLF4-binding site was indeed responsive to KLF4, two pGL3-based luciferase reporter plasmids named BS and BSM were established (Fig. 3d). Then, these plasmids were individually transfected into PC3 cells with or without cisplatin treatment. As shown in Fig. 3e and f, the luciferase activity of BS but not BSM was significantly increased in KLF4 WT PC3 cells in response to cisplatin treatment, and the increase disappeared when KLF4 was knocked out, indicating that the putative binding site was a positive KLF4 binding site in the BIK promoter. In addition, subsequent chromatin immunoprecipitation (ChIP) assays showed that the chromatin fragments corresponding to the putative KLF4 binding sites were specifically present in the anti-KLF4 immunoprecipitates from PC3 cells and the bond was increased during cisplatin treatment, which was then diminished by KLF4 knockout (Fig. 3g-h).

### KLF4 enhances cisplatin-induced apoptosis by regulating BIK expression

**Fig. 4:**
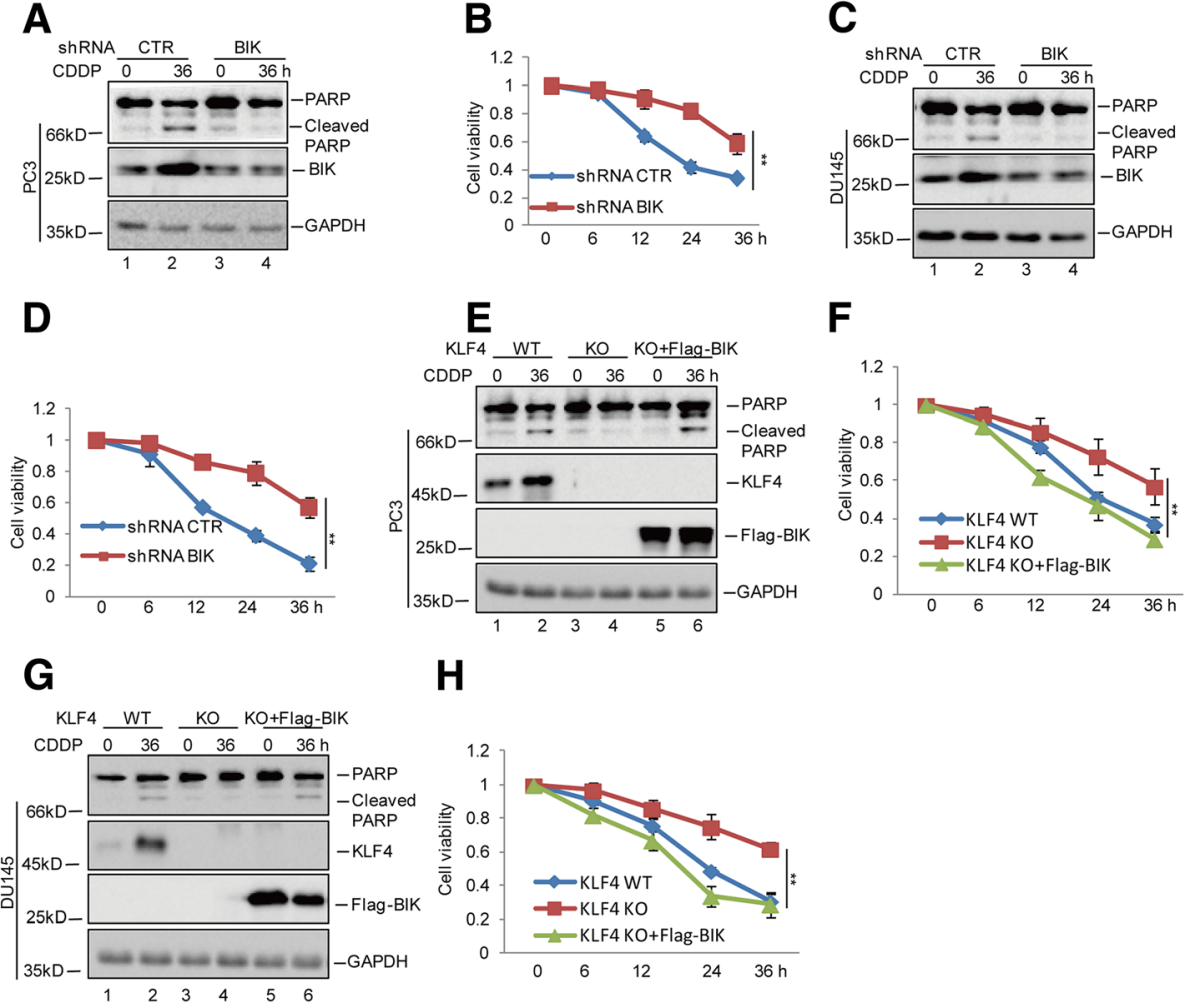
KLF4 promoted cisplatin-induced apoptosis via regulating BIK. **a**-**d** PC3 and DU145 cells with or without BIK knockdown were treated with 20 μM cisplatin for the indicated times. Cell apoptosis was detected by western blotting and cell viability was measured by a CCK8 assay. Data represent the mean ± SD of three independent experiments. **p < 0.01 vs. control. **e**-**h** Flag-BIK was transfected into PC3 and DU145 cells with or without KLF4 knockout and then the cells were treated with 20 μM cisplatin at the indicated times. Cell apoptosis was detected by western blotting and cell viability was measured by a CCK8 assay. Data represent the mean ± SD of three independent experiments. **p < 0.01 vs. control

To determine whether the promotion of chemosensitivity by KLF4 was relied on BIK, we first knocked down BIK in the PC3 and DU145 cells and then treated these cells with cisplatin. Compared with the control cells, the BIK decrease suppressed the cisplatin-induced apoptosis as indicated by a PARP cleavage decrease and promoted the cell viability increase (Fig. 4a-d). Then, BIK was overexpressed in KLF4 KO PC3 and DU145 cells. We found that BIK overexpression reversed the decreased cell apoptosis when KLF4 was lost, indicating the promotion of cisplatin-induced apoptosis by KLF4was dependent on BIK (Fig. 4e-h).

### miR-32-5p inhibits KLF4 expression in prostate cancer

**Fig. 5:**
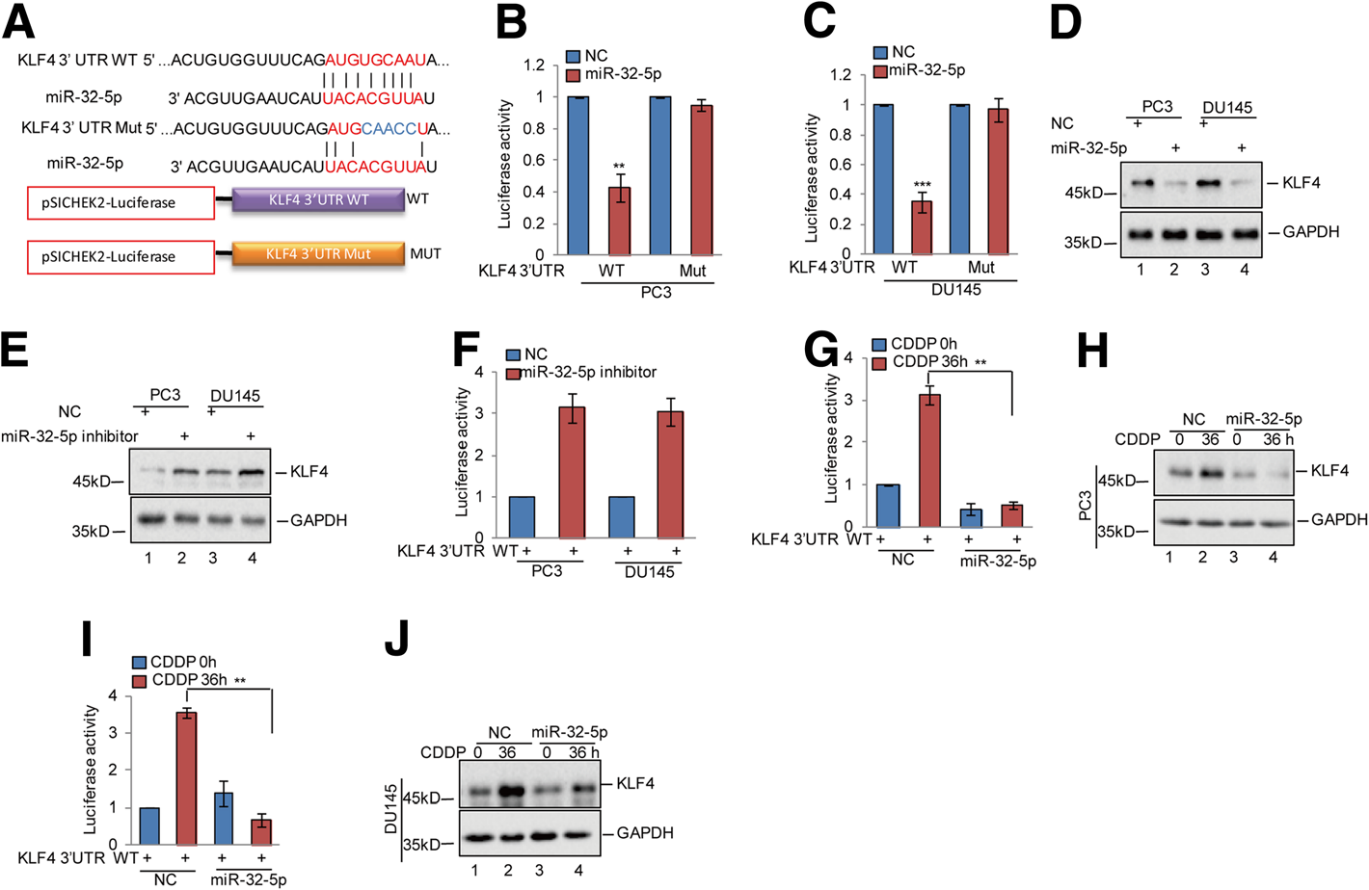
miR-32-5p inhibited KLF4 expression in prostate cancer cells. **a** Potential binding region of miR-32-5p on KLF4 was predicted by TargetScan. The sequences of KLF4 3’UTR containing the wild type miR-32-5p binding site or the mutant were constructed into a pSICHECK2 vector, where the red indicates the mutated region. **b**-**d** The wild type or mutant of KLF4 3’UTR was transfected into PC3 and DU145 cells with or without miR-32-5p overexpression. The luciferase activities were measured. The expression levels of KLF4 were detected by western blotting. Data represent the mean ± SD of three independent experiments. **p < 0.01 and ***p < 0.001 vs. control. **e**-**f** The KLF4 3’UTR was transfected into PC3 and DU145 cells with or without miR-32-5p inhibitor. The luciferase activities were measured. The expression levels of KLF4 were detected by western blotting. Data represent the mean ± SD of three independent experiments. **p < 0.01 vs. control. **g**-**j** The KLF4 3’UTR was transfected into PC3 and DU145 cells with or without miR-32-5p overexpression and then the cells were treated with 20 μM cisplatin at the indicated times. The luciferase activities were measured. The expression levels of KLF4 were detected by western blotting. Data represent the mean ± SD of three independent experiments. **p < 0.01 vs. control

To explore the mechanism underlying cisplatin-induced KLF4 accumulation, we first examined KLF4 mRNA levels by qRT-PCR assay. As shown in Additional file 2: Figure S2A, the mRNA levels of KLF4 were not affected by cisplatin treatment. A large number of studies have shown that miRNAs are important regulators of KLF4 expression in a transcriptionally independent manner [25, 26]. Thus, we sought to identify the miRNAs that were involved in regulating KLF4 expression in response to cisplatin treatment. Through combined RNA sequencing analysis and web-based miRNA resources, we found miR-32-5p was downregulated under cisplatin treatment in prostate cancer cells and was a candidate miRNA that might regulate KLF4 expression (Additional file 2: Figure S2B). To examine whether miR-32-5p regulated KLF4 expression, we constructed the 3’UTR region of KLF4 containing the wild type binding site (WT) or the corresponding binding mutant (Mut) for miR-32-5p into the luciferase reporter system and carried out luciferase assays (Fig. 5a). The results revealed that miR-32-5p suppressed the luciferase activity controlled by the 3’UTR of KLF4 in PC3 and DU145 cells; however, the decrease was abolished when the binding site was mutated (Fig. 5b-c). To examine the influence of miR-32-5p on endogenous KLF4 levels, we treated PC3 and DU145 cells with the corresponding miRNA mimics. As shown in Fig. 5d, the miR-32-5p mimic efficiently suppressed KLF4 protein levels. Furthermore, treatment with the miR-32-5p inhibitor resulted in an increase in KLF4 expression and luciferase activity elevation in prostate cancer cells (Fig. 5e-f). To investigate whether downregulation of miR-32-5p by cisplatin contributed to the KLF4 increase, the 3’UTR of KLF4 was introduced into PC3 and DU145 cells with or without miR-32-5p overexpression and then the cells were treated with cisplatin as indicated. The luciferase activities controlled by the KLF4 3’UTR were measured. Compared with the control cells, cisplatin treatment elevated the luciferase activity under control by the KLF4 3’UTR. However, the elevation was abolished when miR-32-5p was overexpressed (Fig. 5g and h). Similarly, miR-32-5p prevented cisplatin-induced KLF4 expression (Fig. 5i-j). Thus, these data indicate that downregulation of miR-32-5p induced by cisplatin leads to KLF4 upregulation in prostate cancer.

### miR-32-5p downregulates BIK expression via targeting KLF4 in prostate cancer during cisplatin treatment

**Fig. 6:**
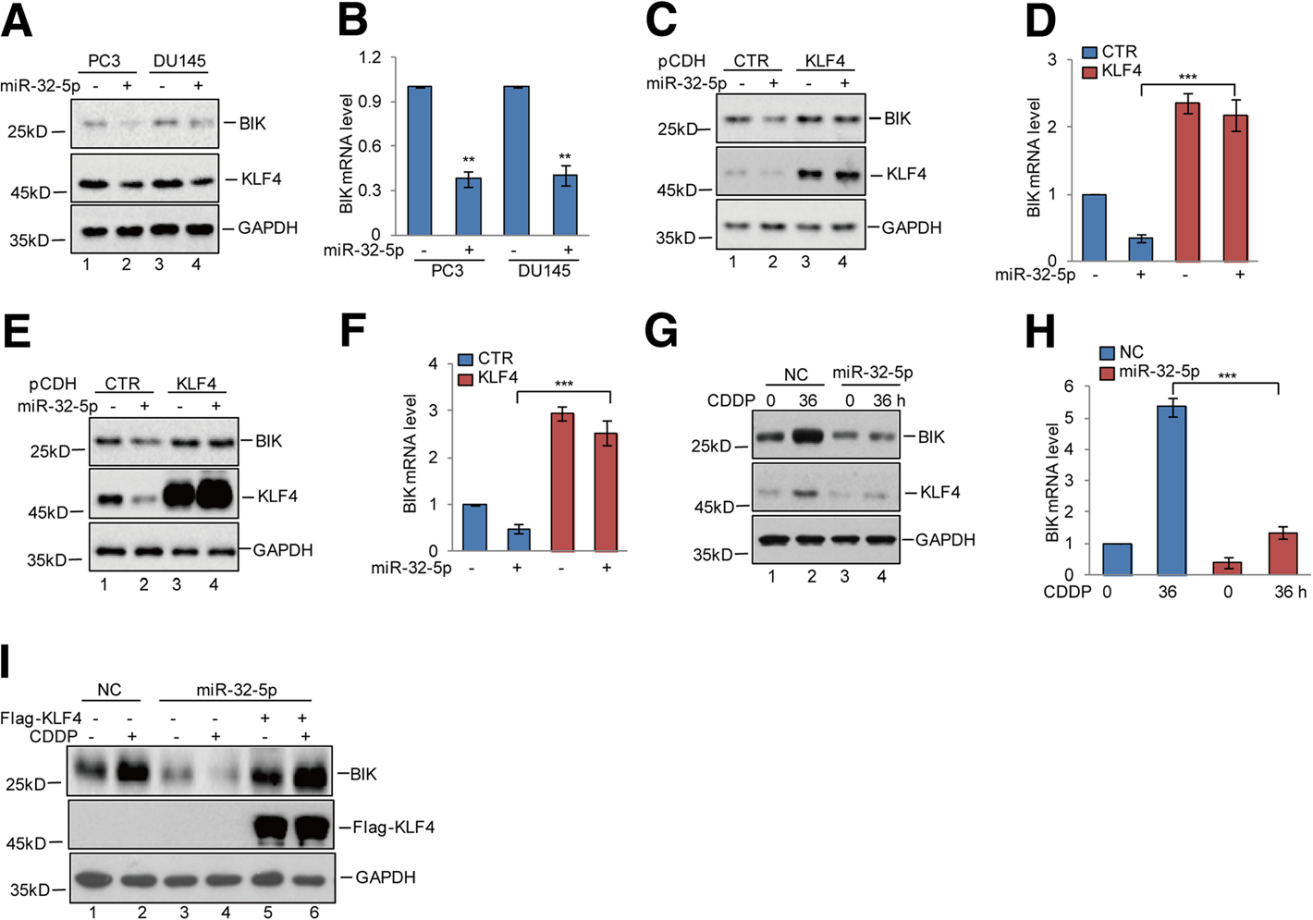
miR-32-5p suppressed BIK expression by targeting KLF4. **a**-**b** miR-32-5p was transfected into PC3 and DU145 cells. Cell lysates were analysed using the indicated antibodies. The mRNA levels of BIK were detected by q-RT-PCR. Data represent the mean ± SD of three independent experiments. **p < 0.01 vs. control. **c**-**f** KLF4 was transfected into PC3 and DU145 cells with or without miR-32-5p overexpression. Cell lysates were analysed using the indicated antibodies. The mRNA levels of BIK were detected by q-RT-PCR. Data represent the mean ± SD of three independent experiments. ***p < 0.001 vs. control. **g**-**h** miR-32-5p was transfected into PC3 cells and then the cells were treated with 20 μM cisplatin at the indicated times. Cell lysates were analysed using the indicated antibodies. The mRNA levels of BIK were detected by q-RT-PCR. Data represent the mean ± SD of three independent experiments. ***p < 0.001 vs. control. **i** Flag-KLF4 was transfected into PC3 cells with or without miR-32-5p overexpression and then the cells were treated with 20 μM cisplatin at the indicated times. Cell lysates were analysed using the indicated antibodies. The mRNA levels of BIK were detected by q-RT-PCR. Data represent the mean ± SD of three independent experiments. ***p < 0.001 vs. control

Given that the gene encoding BIK is upregulated by KLF4, we then examined whether miR-32-5p could affect BIK expression via targeting KLF4. To this end, we first introduced miR-32-5p into PC3 and DU145 cells. The expression levels of BIK were detected by western blotting and q-RT-PCR. We found that overexpression of miR-32-5p decreased BIK expression (Fig. 6a-b). However, the downregulation of BIK expression by miR-32-5p was abolished when KLF4 was exogenously overexpressed (Fig. 6c-f). Subsequently, miR-32-5p was overexpressed in PC3 cells and then the cells were treated with cisplatin. The protein and mRNA levels of BIK were analysed by western blotting and q-RT-PCR. We found that miR-32-5p overexpression suppressed cisplatin-induced BIK expression and the suppression was reversed by KLF4 overexpression (Fig. 6g-i). Taken together, these data indicate that miR-32-5p could inhibit BIK expression via regulating KLF4.

### miR-32-5p contributes to cisplatin-resistance through suppressing the KLF4-BIK axis in prostate cancer

**Fig. 7:**
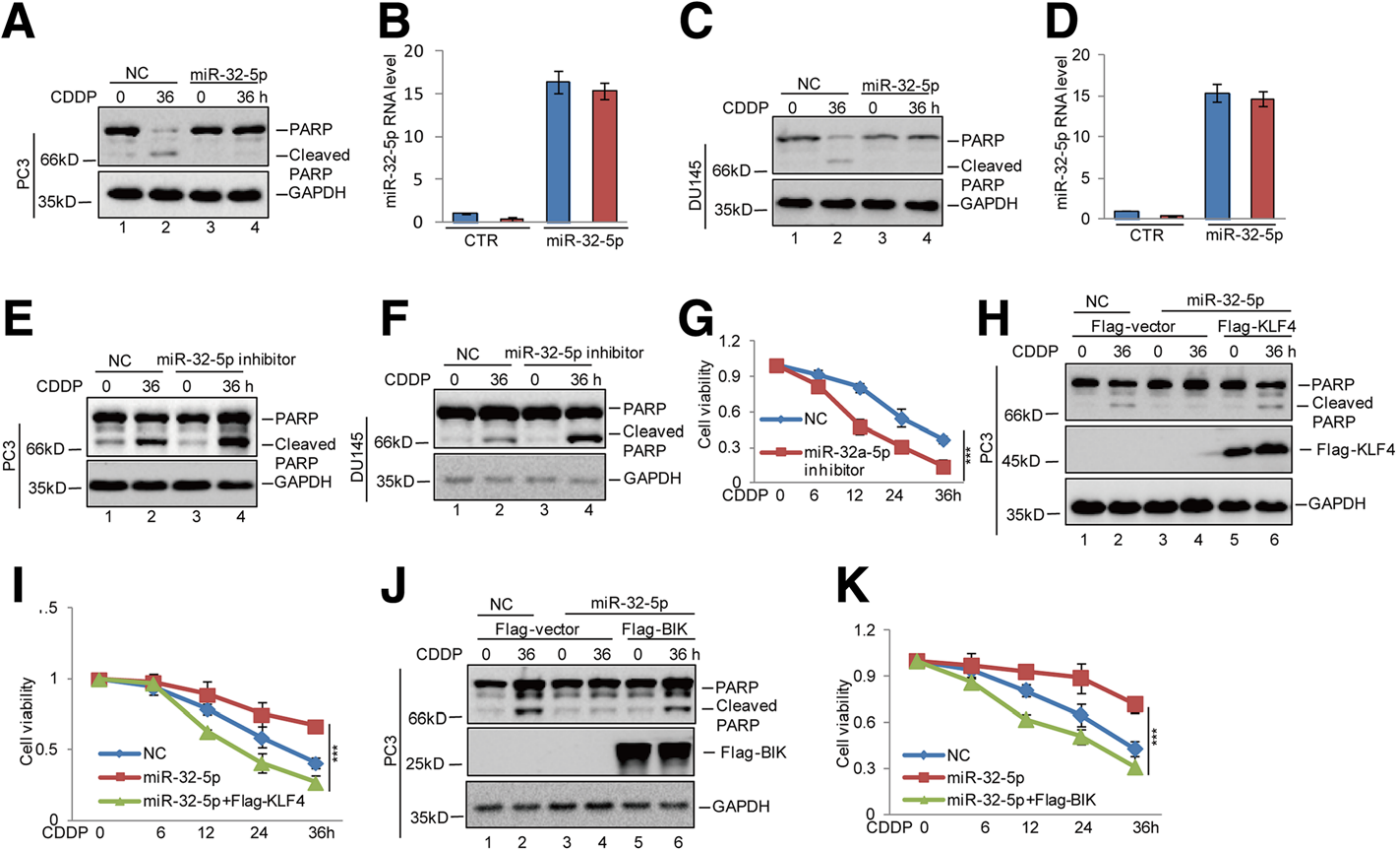
miR-32-5p increased cisplatin-resistance of prostate cancer via inhibition of the KLF4-BIK axis. **a**-**d** miR-32-5p was transfected into PC3 and DU145 cells. The cells were treated with 20 μM cisplatin at the indicated times. Cell apoptosis was analysed by western blotting and cell viability was measured by a CCK8 assay. The expression levels of miR-32-5p were detected by q-RT-PCR. Data represent the mean ± SD of three independent experiments. **e**-**g** miR-32-5p inhibitor was transfected into PC3 and DU145 cells. The cells were treated with 20 μM cisplatin as indicated times. Cell apoptosis was analysed by western blotting. Cell viability was detected by a CCK8 assay. Data represent the mean ± SD of three independent experiments. ***p < 0.001 vs. control. **h**-**k** Flag-KLF4 or Flag-BIK was transfected into PC3 cells with or without miR-32-5p overexpression. The cells were treated with 20 μM cisplatin at the indicated times. Cell apoptosis was analysed by western blotting. Cell viability was detected by a CCK8 assay. Data represent the mean ± SD of three independent experiments. ***p < 0.001 vs. control

Having identified that miR-32-5p could suppress BIK expression via targeting KLF4, We next asked whether miR-32-5p inhibited cisplatin-induced apoptosis via regulating the KLF4-BIK axis. To this end, we first overexpressed miR-32-5p in prostate cancer cells and then the cells were treated with cisplatin. Compared with the control group, upregulation of miR-32-5p decreased cell apoptosis as indicated by a PARP cleavage (Fig. 7a-d). Conversely, inhibition of miR-32-5p enhanced cisplatin-induced cell apoptosis and decreased cell viability (Fig. 7e-g and Additional file 2: Figure S2C-2D). Subsequently, we found that the effects on cell apoptosis and cell viability in response to miR-32-5p overexpression were recovered by KLF4 or BIK overexpression (Fig. 7h-k). Thus, these data suggest that miR-32-5p reduced prostate cancer sensitivity to cisplatin via downregulating KLF4 and BIK expression.

## Discussion

In this study, we found that the protein levels of KLF4 were elevated by cisplatin in prostate cancer cells and increased KLF4 expression enhanced the chemosensitivity to cisplatin. Further mechanistic studies revealed that KLF4 directly bound to the promoter of BIK, facilitating its transcription. Additionally, we also found that the gene encoding KLF4 was a direct target of miR-32-5p. The downregulation of miR-32-5p in response to cisplatin treatment promoted a KLF4 increase and led to a decrease in prostate cancer chemoresistance .

Previous reports showed that KLF4 has a dual role in regulating sensitivity to chemotherapy drugs. In breast cancer and osteosarcoma, upregulation of KLF4 enhanced chemoresistance and inhibited cell apoptosis [27, 28]. However, in a number of cancers, including lung cancer, ovarian cancer and oesophageal squamous cell carcinoma, KLF4 was reported to suppress chemoresistance and enhance cell apoptosis [7, 29, 30]. Similarly, we found that KLF4 was induced by cisplatin and that elevated KLF4 promoted prostate cancer cell apoptosis via transcriptionally upregulating BIK expression.

BIK is a BH3-only, proapoptotic member of the Bcl-2 family of apoptosis regulators, which acts directly on Bcl-2 and Bcl-XL through their common BH3 domain to inactivate their antiapoptotic functions and to provoke apoptosis in a Bax-dependent fashion [31, 32]. Several reports have indicated that BIK expression is increased in response to DNA damage stimuli. Elevated BIK promoted cisplatin and UV-induced mitochondrial apoptosis in colon cancer cells [33]. In head and neck squamous cell carcinoma cells, increased BIK by Bortezomib enhanced cisplatin-induced apoptosis and loss of Bik accelerated murine lymphoma development or rendered lymphoma cells resistant to DNA damaging drugs [34–36]. Consistently, our data showed that BIK was increased in response to cisplatin treatment and we found that KLF4 upregulated the expression of BIK in prostate cancer cells and promoted cisplatin-induced BIK expression.

In contrast to the response to several other stimuli where the KLF4 mRNA level was increased, we did not observe distinct changes of KLF4 mRNA in response to cisplatin treatment. Accumulating evidence has suggested that several miRNAs are involved in regulating KLF4 expression such as miR-103, miR-10b and miR-29a [25, 26, 37–39]. Our data showed that miR-32-5p inhibited KLF4 expression in prostate cancer cells. MiR-32-5p has been previously reported to induce multidrug resistance in hepatocellular carcinoma via the PI3K/Akt pathway [40, 41]. Similarly, we found that miR-32-5p enhanced the chemoresistance of prostate cancer and inhibited cisplatin-induced apoptosis via reducing KLF4-BIK signalling pathway activity. In addition, our data also suggest that the expression levels of miR-32-5p were downregulated in response to cisplatin treatment in prostate cancer. However, the molecular mechanism involved needs to be studied in the future.

## Conclusions

Taken together, our data suggest that KLF4 is an essential regulator in cisplatin-induced apoptosis, and the miR-32-5p-KLF4-BIK signalling axis plays an important role in cisplatin sensitivity of prostate cancer.

## Additional files

Additional file 1: **Figure S1.** (A) KLF4 WT or KO PC3 cells were treated with 20 μM cisplatin at the indicated times. Gene expression profiles were obtained by RNA sequencing analysis. The number of changed genes (fold change > 2) was analysed. (B-C) Significantly enriched GO terms are listed. (TIF 521 kb)

Additional file 2: **Figure S2.** (A-B) The RNA levels of KLF4 and miR-32-5p were analysed by q-RT-PCR in prostate cancer cells with cisplatin treatment at the indicated times. (C-D) The expression levels of miR-32-5p were detected by q-RT-PCR. (TIF 239 kb)

## Abbreviations

BIK: Bcl2-Interating Killer CCK8 cell counting kit ChIP Chromatin Immunoprecipitation CDDP cisplatin KLF4 KLF-like factor4
