# Correction: KLF4, a miR-32-5p targeted gene, promotes cisplatin-induced apoptosis by upregulating BIK expression in prostate cancer

**DOI:** 10.1186/s12964-023-01198-x

**Published:** 2023-06-22

**Authors:** Lu Zhang, Xiaojie Li, Yulin Chao, Ruiping He, Junqiang Liu, Yi Yuan, Wenzhi Zhao, Chuanchun Han, Xishuang Song

**Affiliations:** 1 Department of Urology of the First Affiliated Hospital & Institute of Cancer Stem Cell, Dalian Medical University, Dalian, Liaoning 116044 People’s Republic of China; 2 Department of Orthopedics, Second Affiliated Hospital, Dalian Medical University, Dalian, 116044 China; 3 College of Stomatology, Dalian Medical University, Dalian, 116044 China

**Correction: Cell Commun Signal 16, 53 (2018)**

**https://doi.org/10.1186/s12964-018-0270-x**

Following publication of the original article [1], the authors identified that the western blot bands of KLF4 and GAPDH in Fig. 1L, and the band of KLF4 in Fig. 5H were incorrect. The correct figures are supplied in this article. Corrections to these bands do not change the conclusion to the paper. The authors apologize for the error.

**Fig. 1:**
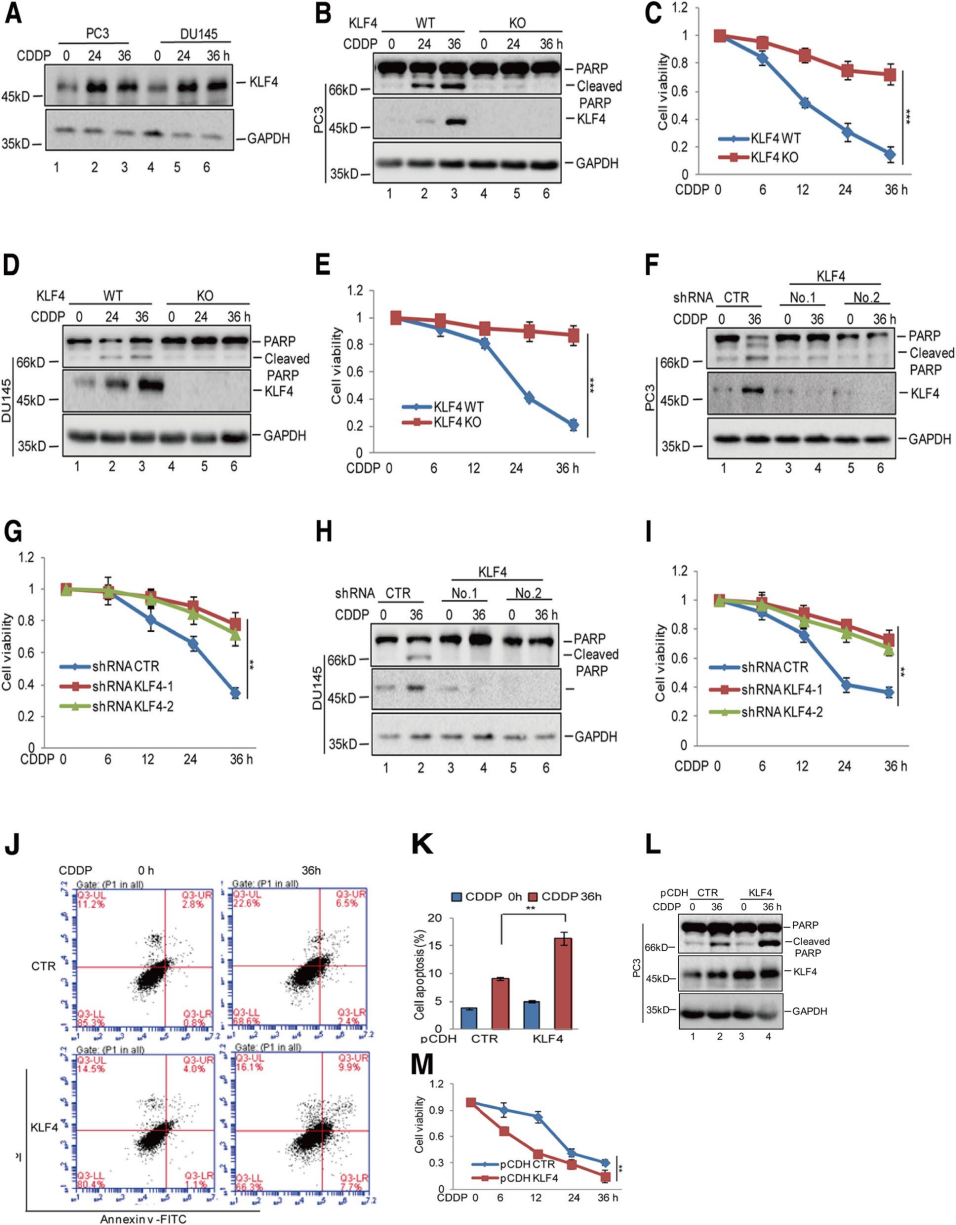
KLF4 enhanced cisplatin-induced apoptosis in prostate cancer cells. **a** PC3 and DU145 cells were treated with 20 μM cisplatin (CDDP) at the indicated times. The protein levels of KLF4 were analysed by western blotting. **b**-**e** PC3 and DU145 cells with or without KLF4 knockout (KO) were treated with 20 μM cisplatin at the indicated times. Cell apoptosis was detected by western blotting. Cell viability was measured by a CCK8 assay. Data represent the mean ± SD of three independent experiments. ***p < 0.001 vs. control. **f**-**i** PC3 and DU145 cells with or without KLF4 knockdown were treated with 20 μM cisplatin at the indicated times. Cell apoptosis was detected by western blotting and cell viability was measured by a CCK8 assay. Data represent the mean ± SD of three independent experiments. **p < 0.01 vs. control. **j**-**m** PC3 cells with or without KLF4 overexpression were treated with 20 μM cisplatin as indicated. Cell apoptosis was analysed by flow cytometer and western blotting. Cell viability was detected by a CCK8 assay. Data represent the mean ± SD of three independent experiments. **p < 0.01 vs. control

**Fig. 5:**
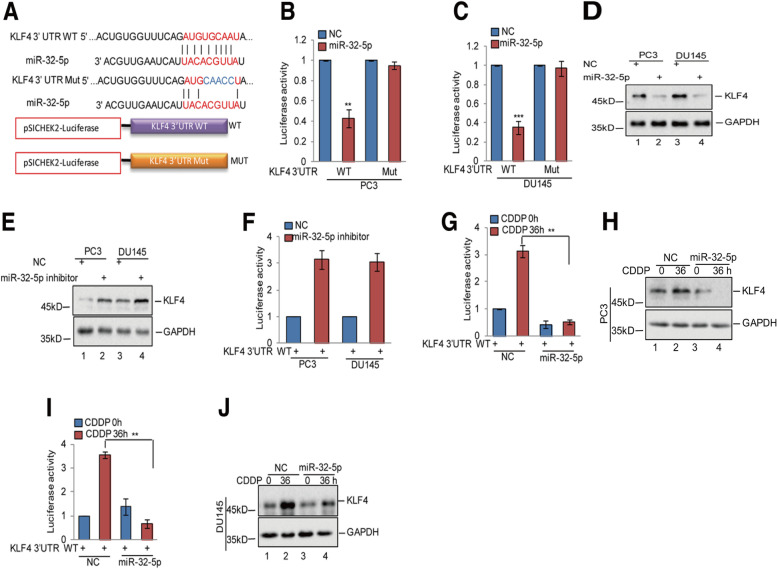
miR-32-5p inhibited KLF4 expression in prostate cancer cells. **a** Potential binding region of miR-32-5p on KLF4 was predicted by TargetScan. The sequences of KLF4 3’UTR containing the wild type miR-32-5p binding site or the mutant were constructed into a pSICHECK2 vector, where the red indicates the mutated region. **b**-**d** The wild type or mutant of KLF4 3’UTR was transfected into PC3 and DU145 cells with or without miR-32-5p overexpression. The luciferase activities were measured. The expression levels of KLF4 were detected by western blotting. Data represent the mean ± SD of three independent experiments. **p < 0.01 and ***p < 0.001 vs. control. **e**–**f** The KLF4 3’UTR was transfected into PC3 and DU145 cells with or without miR-32-5p inhibitor. The luciferase activities were measured. The expression levels of KLF4 were detected by western blotting. Data represent the mean ± SD of three independent experiments. **p < 0.01 vs. control. **g**-**j** The KLF4 3’UTR was transfected into PC3 and DU145 cells with or without miR-32-5p overexpression and then the cells were treated with 20 μM cisplatin at the indicated times. The luciferase activities were measured. The expression levels of KLF4 were detected by western blotting. Data represent the mean ± SD of three independent experiments. **p < 0.01 vs. control
