# Supplementary figures and images for: KLF4, a miR-32-5p targeted gene, promotes cisplatin-induced apoptosis by upregulating BIK expression in prostate cancer
Source: Cell Commun Signal. 2018 Sep 3;16:53. doi: 10.1186/s12964-018-0270-x (PMC6122640; doi:10.1186/s12964-018-0270-x)

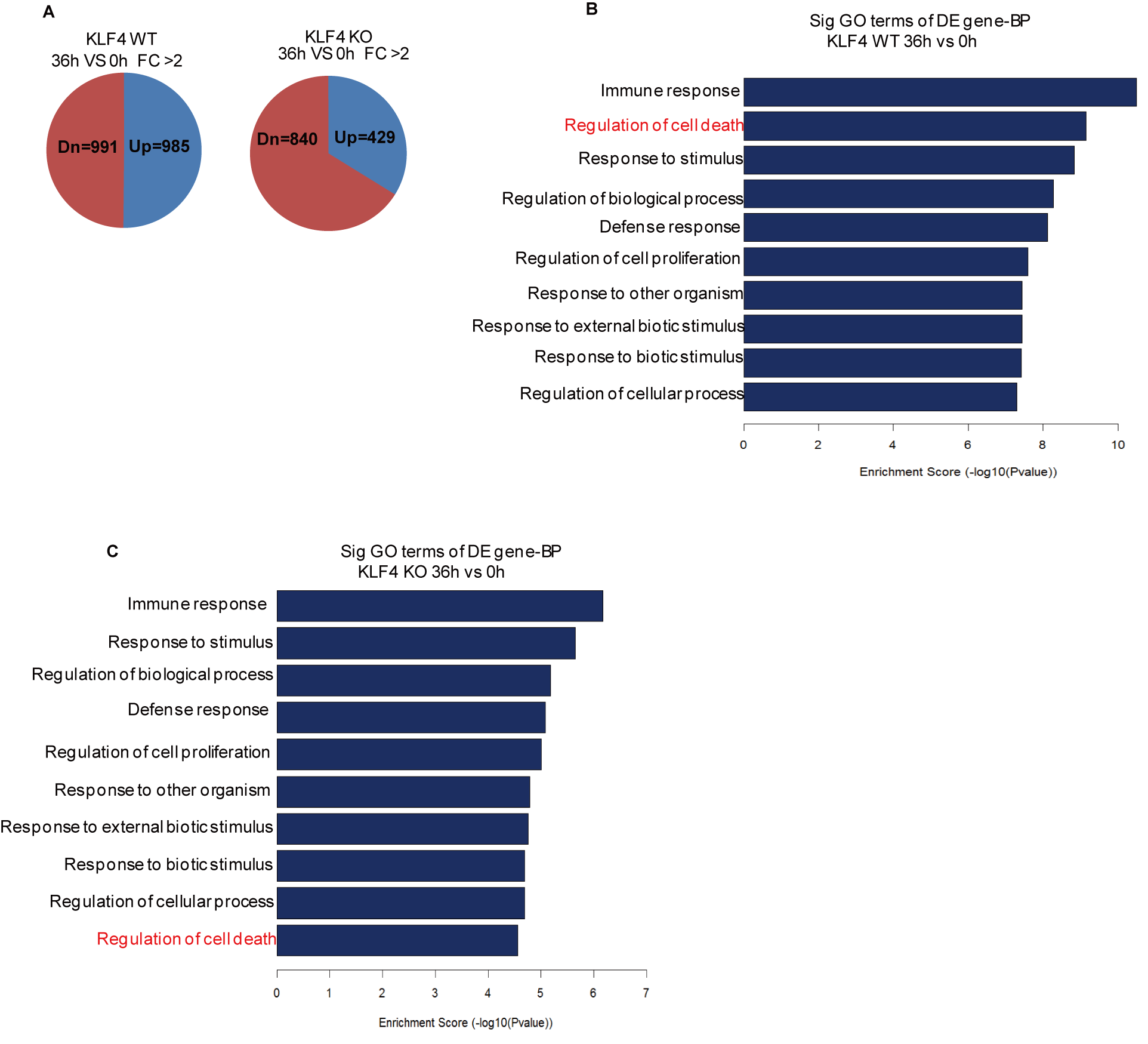

Supplement: Supplementary file 1 — Figure S1. (A) KLF4 WT or KO PC3 cells were treated with 20 μM cisplatin at the indicated times. Gene expression profiles were obtained by RNA sequencing analysis. The number of changed genes (fold change > 2) was analysed. (B-C) Significantly enriched GO terms are listed. (TIF 521 kb) [file 12964_2018_270_MOESM1_ESM.tif]

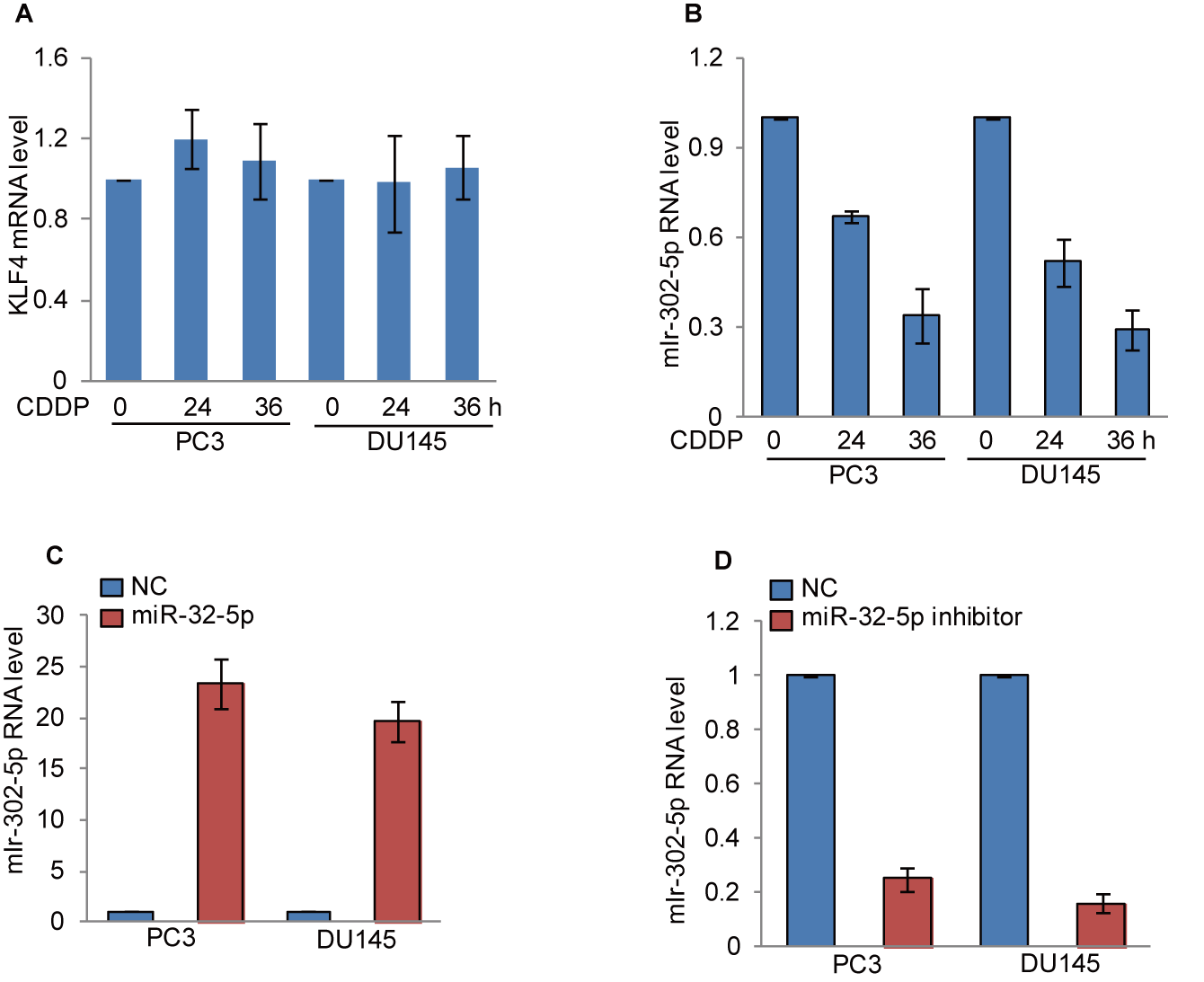

Supplement: Supplementary file 2 — Figure S2. (A-B) The RNA levels of KLF4 and miR-32-5p were analysed by q-RT-PCR in prostate cancer cells with cisplatin treatment at the indicated times. (C-D) The expression levels of miR-32-5p were detected by q-RT-PCR. (TIF 239 kb) [file 12964_2018_270_MOESM2_ESM.tif]
